# Supplementary material for: Dynalign II: common secondary structure prediction for RNA homologs with domain insertions
Source: Nucleic Acids Res. 2014 Nov 21;42(22):13939–48. doi: 10.1093/nar/gku1172 (PMC4267632; doi:10.1093/nar/gku1172)
Supplement: SUPPLEMENTARY DATA [file supp_gku1172_nar-02021-z-2014-File012.zip › manual/RNA_class/html/design_8h.html]

RNAstructure Classes: E:/RNAstructure/RNA\_class/design.h File Reference

- Main Page
- Classes
- Files

- File List
- File Members

# E:/RNAstructure/RNA\_class/design.h File Reference

`#include "../RNAstructure/RNA_class/RNA.h"`  
`#include "../RNAstructure/src/random.h"`  
`#include <cstdlib>`  
`#include <vector>`  

|  |  |
| --- | --- |
|  |  |
| Classes | |
| class | design |
| Defines | |
| #define | DESIGN\_H |
| Functions | |
| int | pow (const int i, const int j) |

---

## Define Documentation

#define DESIGN\_H

---

## Function Documentation

|  |  |  |  |  |
| --- | --- | --- | --- | --- |
| int pow | ( | const int | *i*, |  |
|  |  | const int | *j* |  |
|  | ) |  |  | `[inline]` |

---

Generated on Mon Apr 23 16:48:03 2012 for RNAstructure Classes by 
 1.5.7.1
